# Assessing the cost of providing a prevention of mother-to-child transmission of HIV/AIDS service in Ethiopia: urban-rural health facilities setting

**DOI:** 10.1186/s12913-019-3978-4

**Published:** 2019-03-06

**Authors:** Elias Asfaw Zegeye, Josue Mbonigaba, Sylvia Kaye, Benjamin Johns

**Affiliations:** 1 Economics Department, University of KwaZulu-Natal, Durban, South Africa; 2 Abenezer Consulting PLC, Economic Evaluation and Health Care Financing Division, Addis Ababa, Ethiopia; 3 Public Administration and Economics Department, Durban University of Technology, Durban, South Africa; 4 Abt Associates Inc, Bethesda, USA

**Keywords:** Cost analysis, Unit cost, PMTCT service packages, Resource ingredients, Micro-costing, Urban, Rural and Ethiopia

## Abstract

**Background:**

While local context costing evidence is relevant for healthcare planning, budgeting and cost-effectiveness analysis, it continues to be scarce in Ethiopia. This study assesses the cost of providing a prevention of mother-to-child transmission of HIV/AIDS (PMTCT) service across heterogeneous prevalence (high, low) and socio-economic (urban, rural) contexts.

**Methods:**

A total of 12 health facilities from six regions in Ethiopia were purposively selected from the latest 2012 antenatal sentinel HIV prevalence report. Six health facilities with the highest HIV prevalence (8.1 to 17.3%) in urban settings and six health facilities with the lowest prevalence (0.0 to 0.1%) in rural settings were selected. A micro-costing approach was applied to identify, measure and value resources used for the provision of a comprehensive PMTCT service. The analysis was conducted across different PMTCT service packages. We also estimated national costs in urban and rural contexts.

**Results:**

The average cost per pregnant woman-infant pair per year (PPY) ranged from ETB 6280 (USD 319) to ETB 21,620 (USD 1099) in the urban high HIV prevalence health facilities setting. In rural low HIV prevalence health facilities, the cost ranged from ETB 4323 (USD 220) to ETB 7539 (USD 383).PMTCT service provision in urban health facilities costs more than twice the cost in rural health facilities. The average cost per PPY in an urban setting was more than double the cost in a rural setting due to the higher cost of inputs and possible inefficiencies (although there were a higher number of visits). Consumables (including antiretroviral drugs) and infrastructure were the major cost drivers in both the urban and rural health facilities. Among PMTCT service components, anti-retroviral treatment Option B+ follow-up and counselling accounted for the highest proportion of costs, which ranged from 58 to 72%. Nationally, at the current coverage, the cost of PMTCT service was USD 6 million and USD 3 million in urban and rural settings, respectively.

**Conclusions:**

The analysis suggests that resources used for PMTCT service packages varied across health facilities and HIV prevalence contexts. Providing PMTCT service in the high HIV prevalence urban health facilities costs more than in the rural facilities. Context-specific costing was vital to provide locally sensitive evidence for health service management and priority setting.

**Electronic supplementary material:**

The online version of this article (10.1186/s12913-019-3978-4) contains supplementary material, which is available to authorized users.

## Background

Since the 1980s, HIV/AIDS has struck every aspect of human life. HIV/AIDS has affected the household economy, agricultural productivity, business, education, the health sector and the economic development of countries across the globe. Globally in 2012, 35 (32.2 to 38.8) million people were living with HIV/AIDS, 2.3 (1.9 to 2.7) million people were newly infected with HIV, while 1.6 (1.4 to 1.9) million died of HIV/AIDS [1]. In 2015, 52% (19 million) of the 36.7 million people with HIV/AIDS worldwide were living in the eastern- and southern African regions [2]. Since 2010, an increase in the number of people living with HIV, as well as a decrease in new HIV infections and AIDS-related deaths have been reported [1–3]. Infections among children decreased by 35% in 2012 relative to 2009 [2], even though new HIV infections in children had been projected to account for 13% of the global total infections during the period 2005–2015 [4].

In Ethiopia, the HIV epidemic has progressed steadily over the last two decades. According to the single point estimate by the Federal HIV/AIDS Prevention and Control Office [5], there were 541,414 new diagnoses of HIV-positive pregnant women and 98,283 HIV-positive births from 2004 to 2010. In the general population, the past four surveillance rounds indicated the highest HIV prevalence of 5.2% in urban areas and the lowest of 0.8% in rural areas [6–8]. Recent HIV prevalence projections (2011–2016) indicate that 2,595,479 patients were in need of antiretroviral therapy (ART) during the period 2011–2016,with 65 and 35% of ART needs reported in urban and rural settings, respectively [9]. Within the same period, out of a projected total of 25,220,323 orphans in urban and rural areas, 4,554,568 orphans would be as a result of AIDS. Including the direct vertical transmission of HIV from mother to child, a total of 109,105 new HIV infections was estimated in the country in the period 2011–2016 [9]. Although these projections suggest a high impact of HIV/AIDS in the country overall, it is worth noting that the last four rounds of surveillance and national demographic surveys reports revealed higher HIV prevalence in urban areas than in rural settings [6–8, 10]. The most recent sentinel surveillance results also showed higher HIV prevalence among pregnant women in the urban antenatal care health facilities [6].

PMTCT interventions have been playing a cross-cutting role, making an impact on three of the previously targeted international Millennium Development Goals (MDGs). These goals were related to the reduction of child mortality, maternal health and HIV/AIDS. PMTCT is also having an effect on the current Sustainable Development Goals (SDG)/ Universal Health Coverage (UHC) targets. Despite their expected role in the international development agenda, PMTCT services have not been widely implemented or utilised by eligible pregnant women [11]. According to the Ethiopian Federal Ministry of Health, in the period 2014–2016, the national uptake of the PMTCT service was 57% [11]. This was very low as compared to the 90% coverage in Ghana, Zambia, Namibia and Botswana [1]. In 2012, Ethiopia was also one of the 22 priority countries achieving less than 50% of antiretroviral treatment coverage for HIV-positive pregnant women [1]. Furthermore, there has been a dearth of costing studies to guide policy decision making in this country [12].

With the elimination of mother-to-child transmission (eMTCT) of HIV infection as a top priority, one piece of crucial information needed by policy-makers and funding organisations concerns the best use of available budgets. Another critical piece of related information includes costing and efficiency analysis for budgeting and cost-effective decisions and the existing literature has not exhaustively analysed these issues [13, 14]. Limited evidence in this respect is likely to lead to inefficient use of resources [12].

Ethiopia, particularly, has faced a dearth of costing evidence to inform PMTCT programme implementation. To the best of our knowledge, Kombe et al. [15], Bikilla et al. [16] and Federal HIV/AIDS Prevention and Control Office (FHAPCO) [17] provide the only studies that report evidence-related to the costs of HIV/AIDS services. Kombe and colleagues [15] surveyed six regional hospitals and estimated the scale-up cost for antiretroviral treatment, voluntary counselling and testing and PMTCT services. Bikilla et al. [16] reported the average cost estimates for the antiretroviral treatment of HIV services at a hospital located in the southern part of the country. More recently, a national antiretroviral treatment costing survey was conducted by FHAPCO [17],although it focused on outpatient costs only, excluding inpatient-related resources. None of these studies addressed the cost of PMTCT services across heterogeneous HIV prevalence contexts (urban versus rural) applying a bottom-up ingredient costing approach. This study aims to fill this gap.

### Overview of the PMTCT service

The government’s response to prevent mother-to-child HIV transmission can best be understood within the broad context of the health sector strategy in Ethiopia. The country introduced the Health Sector Development Program IV (HSDP IV) plan as a health chapter of the national Growth and Transformation Plan (GTP) during the period 2011 to 2015 [18] and launched a new Health Sector Transformation Plan (HSTP) for 2015/16–2019/20 [11].

**Fig. 1:**
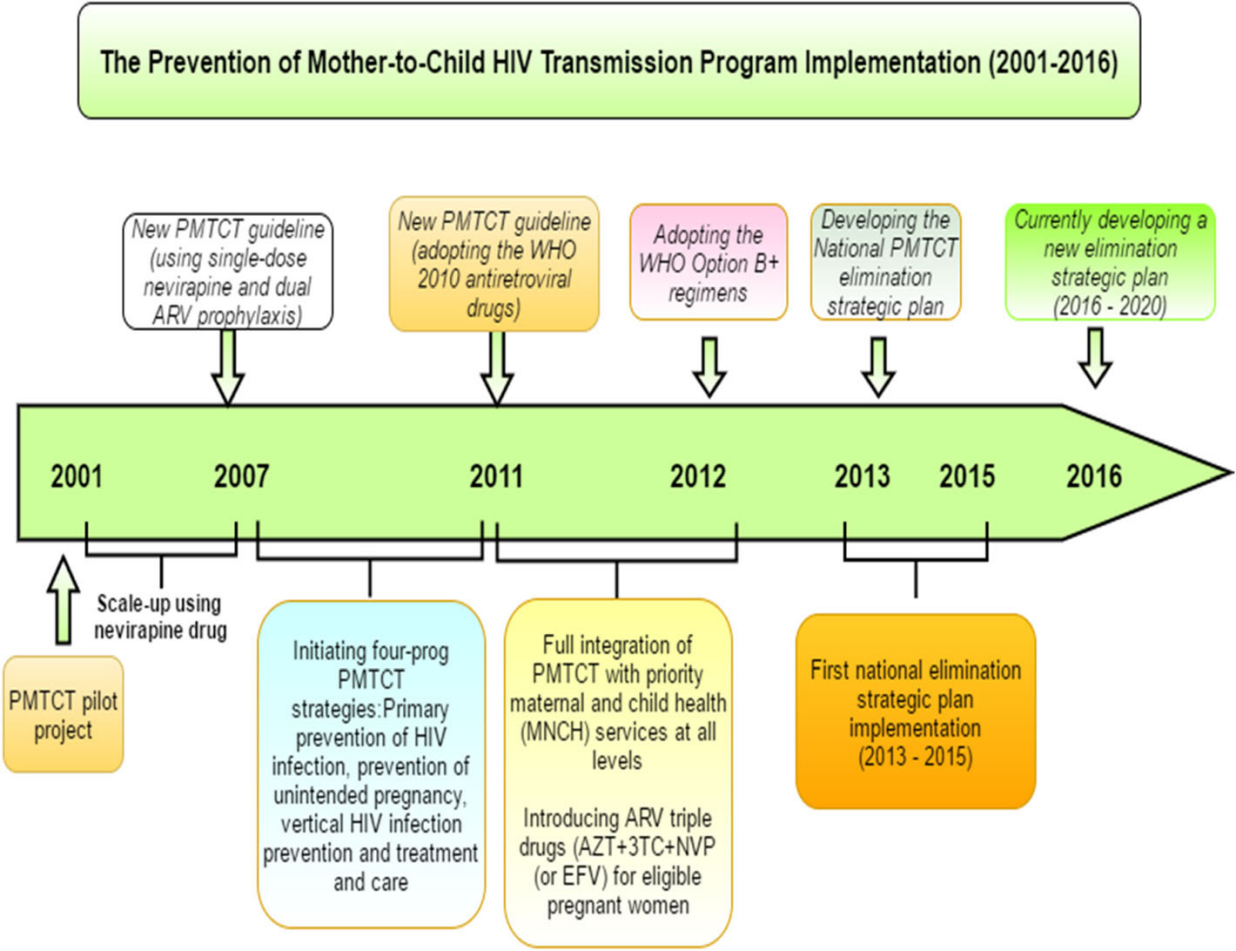
PMTCT programme implementation (2001–2016) in Ethiopia. Own design

The programme started in 2001 at four pilot hospitals in Ethiopia. Thereafter, the intervention was scaled up to 14 health facilities using a single dose Nevirapine (NVP) regimen through a project called HAREG. HAREG was the first pilot project for PMTCT and operated through a close partnership between the FHAPCO and the President’s Emergency Plan for AIDS Relief (PEPFAR). The PMTCT service was further integrated with the maternal and child health programmes in 10 hospitals and 13 health centres [19]. Two PMTCT treatment options, single dose NVP and dual antiretroviral (ARV) prophylaxis, were started in 2007. Subsequently, a comprehensive four-pronged PMTCT strategy was adopted [20, 21] consisting of prevention of HIV infection; prevention of unintended pregnancy; direct (vertical) transmission prevention of HIV from mothers to infants; and treatment, care and support. The detail of the programme rollout in Ethiopia is summarised in Fig. 1.

**Fig. 2:**
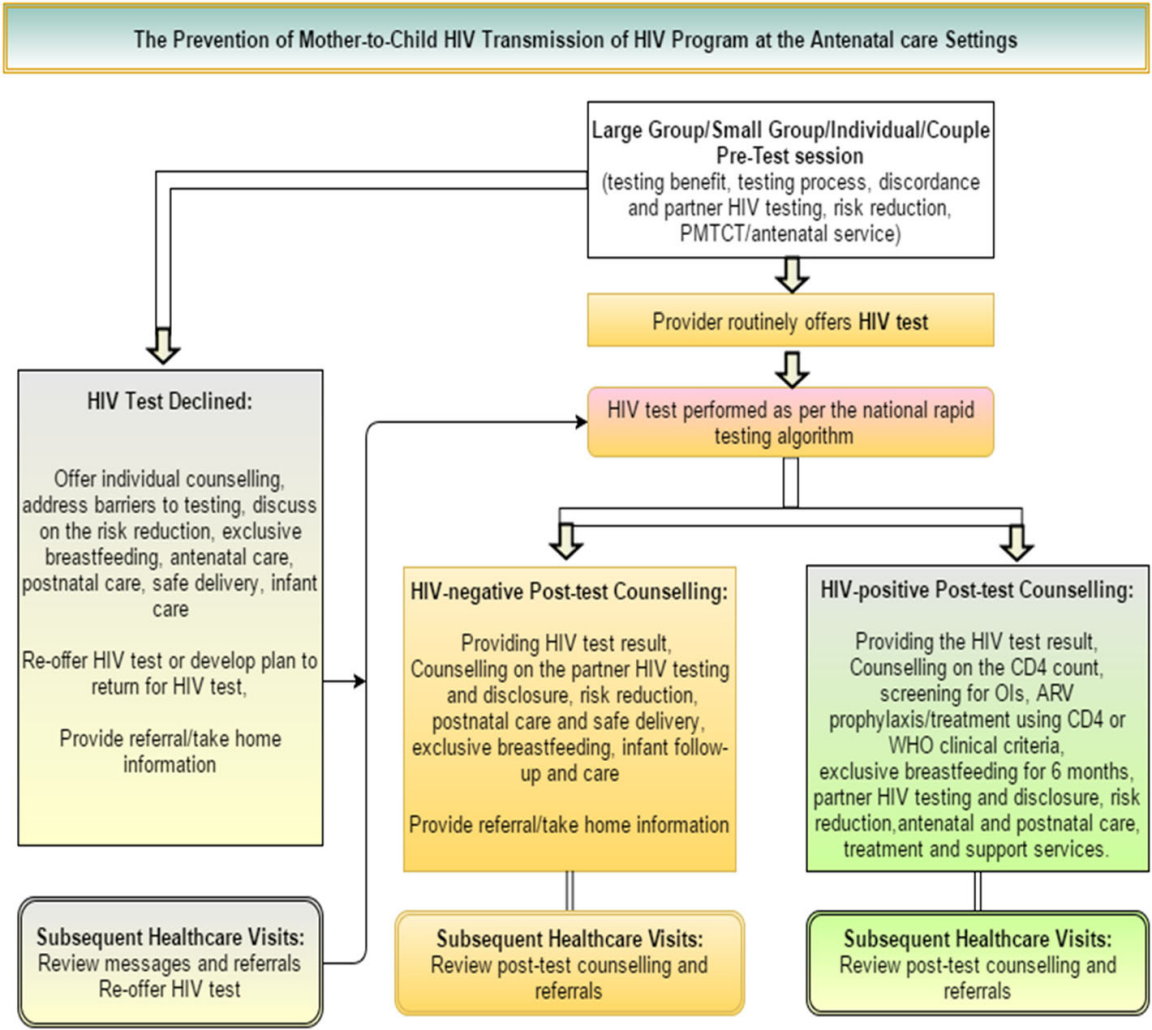
The PMTCT programme different service packages in Ethiopia. Federal HIV/AIDS prevention and control office PMTCT guideline, 2007 and 2011

PMTCT services were provided during antenatal care (ANC) follow-up and at labour and delivery units as well as during post-delivery care. In accordance with the guideline, all pregnant women coming for ANC visits are requested to agree to HIV pre-testing services through opt-out counselling (allowing them to refuse) [20, 21]. If the woman agrees, HIV-testing is conducted. If she does not, she will be offered another opportunity to do the test during the next ANC visit. If the pregnant woman is HIV-positive, she will pass through post-test HIV-positive counselling, which includes services such as providing HIV test results and information on the importance of antiretroviral prophylaxis, requesting HIV testing and counselling for the partner and providing relevant information on exclusive breastfeeding. During follow-up visits, the pregnant woman will initiate ARV prophylaxis treatment. A CD4 count will be conducted every six months. Following the national Health Sector Development Plan (HSDP), in 2012 the Ethiopian government launched the Accelerated Elimination Strategic Plan [22]. In the same year, the World Health Organization (WHO) treatment option B+ was adopted [23]. The detail of the programme flow is explained in Fig. 2.

## Methods

The analysis employed a micro-costing ingredient approach from the perspective of the health care provider. According to the micro-costing, bottom-up approach, the following costing procedures were followed: framing the cost analysis; developing the cost inventory; quantifying costs; and calculating summary measures [24]. The data collection commenced in August 2015 and ended in September 2015.

### Costing methods

As suggested by Drummond et al. [25], ingredient items were identified, estimated and valued with their corresponding unit prices. As the first step, resource ingredients, such as health care professionals’ time, medical supplies, medicines, equipment, transportation, infrastructure and training costing data were identified. Applying an interview costing guide, expert interview sessions were conducted with the PMTCT focal person at the surveyed health facilities to determine yearly quantities utilised. The valuations of resources were estimated considering the average cost data from the Pharmaceutical Fund and Supply Agency (PFSA) in which prices were reported in the 2014 Ethiopian birr (ETB). These prices were converted to the United States dollar (USD) at the 2014 weighted exchange rate (1 US$ =19.6705 ETB) [26].

The costing analysis employed the following formula: C = L + Co +E +I, where C = cost, L = labour, Co = consumables, E = equipment and I = infrastructure. Prices were adjusted for inflation, employing International Monetary Fund (IMF) estimates [27]. An inflation conversion factor and 2014 as a base year were used. Additional costing data, related to the programme management and training costs, were collected at the regional health bureaus. We used a 3% annual discount rate in accordance with standard practice in the literature [28].

Each resource ingredient (fixed and variable) was measured per pregnant woman-infant pair per year (PPY). Costing was reported per PPY for urban and rural settings to be able to compare differences across these two settings. In addition, national estimates of the number of HIV-positive pregnant women in need of treatment were based on the Federal Ministry of Health/ Ethiopian Public Health Institute’s HIV related estimates and projection database [9]. This number, in turn, was used to estimate the national costs of PMTCT services in rural and urban settings.

### Study site selection

The Ethiopian Public Health Institute (EPHI) identified 117 antenatal-based sentinel surveillance sites in different geographical regions in the country [6]. Of the total 117 ANC sites, six urban health facilities, with the highest HIV prevalence among pregnant women ranging from 8.1 to 17.3%, and six rural health facilities, with a prevalence rate ranging from 0.01 to 0.1%, were selected. Heterogeneity in both HIV prevalence and urban and rural locations was used as criteria to select 12 health facilities, which are located in six regions across Ethiopia. The choice of highest and lowest HIV prevalence health facilities (ANC-PMTCT sites) was informed by the need to obtain a clear picture on differences in costs. Urban areas recorded higher HIV prevalence, which is attributed to the fact that urban areas host most-at-risk population groups, notably sex-workers, drug injectors, long-distance truck drivers, members of the armed forces and men who have sex with men. The profile of the 12 health facilities in terms of regional origin, rural and urban context and level of HIV prevalence, is reported in Table 1.

**Table 1:** List of costing study sites, including the HIV prevalence and health facility location

Surveyed health facilities	Region	HIV Prevalence at the health facility (%)	Direction from centre	Setting
Bahir Dar Hospital	Amhara	17.3	Northern Ethiopia	Urban
HiwotFana Hospital,	Harrari	8.8	Eastern Ethiopia	Urban
DileChora Hospital	Dire Dawa	8.1	Eastern Ethiopia	Urban
AFRTH Hospital	Addis Ababa	8.7	Addis Ababa	Urban
Soddo Health Center	SNNPR	8.8	Southern Ethiopia	Urban
Teklehaimanot Health Center	Addis Ababa	8.8	Addis Ababa	Urban
Limuseka Health Center	Oromiya	0.02	Western Ethiopia	Rural
Daddim Health Center	Oromiya	0.04	Western Ethiopia	Rural
Toke Health Center,	Oromiya	0.01	Western Ethiopia	Rural
Chewaka Health Center	Oromiya	0.05	Western Ethiopia	Rural
Kokosa Health Center	Oromiya	0.02	Eastern Ethiopia	Rural
Hasange Health Center	Harrari	0.03	Eastern Ethiopia	Rural

Source: Antenatal care (ANC) sentinel HIV/AIDS PMTCT surveillance report [6]: Ethiopia Public Health Institute (EPHI). Report on the 2012 round Antenatal case based sentinel surveillance in Ethiopia. Addis Ababa. Ethiopia; 2014

The HIV prevalence at ANC sentinel surveillance site was measured as the number of women using antenatal service, who are HIV-positive, as a proportion of total number of women who are attending/using antenatal services during the report period (2012)

### PMTCT service packages

According to PMTCT service protocols, PMTCT service packages cover HIV pre-test counselling, HIV testing, HIV-negative post-test counselling, HIV-positive post-test counselling, ART (Option B+) treatment initiation and counselling, Option B+ follow-up, drug refilling and counselling (for the mother and infant pair), CD4 count (blood sample) service, early infant diagnosis (EID) service, programme management, including the community-based Health Development Army and referral linkage with care and support. Resource ingredients were mapped and estimated for the individual PMTCT service component. Service utilisation data were collected from the Regional Health Bureaus (RHBs) monitoring and evaluation departments. Additional health facility records and performance data were also retrieved from routine Health Management Information System (HMIS) 2013 and 2014 databases. Supplementary national PMTCT service data, such as infrastructure building costs, equipment and medical supplies, were collected from the Federal Ministry of Health (FMoH), the Pharmaceutical Fund and Supply Agency (PFSA) and the FHAPCO.

### Average cost analysis

Detailed average cost analysis steps were conducted as follows:

- *Labour cost:* The national public sector health professionals’ salary scale was obtained from the FMoH Human Resource Development and Management directorate [29]. Each salary category consisted of salary scale levels *(from 0 to 10)* depending on the years of working experience and education qualification. The median salary scale was calculated for each health care cadre. In addition to the basic salary, additional labour benefit packages, such as housing and night duty allowances were estimated from the expert interview sessions. The net working days was calculated, after deducting annual leave, national days and the sick leave allowance in line with the Ethiopian civil servant protocol (Federal Civil Servant Proclamation, no 262/2002 [30]). Staff time spent on PMTCT services was determined by asking team leaders/managers the proportion of time each staff/cadre spends on a specific PMTCT service. The study applied that proportion to the daily rate of that category of staff.
- Supplies cost: First, in the surveyed health facilities, a list of relevant supplies used for the PMTCT service was compiled in consultation with health care professionals, including pharmacists and storekeepers. A total of 12 PMTCT focal persons and 10 pharmacists were interviewed to identify the supplies on the list. The complementary unit of the costing measure for identified resources was retrieved from the National Pharmaceutical Fund and Supply Agency (PFSA) national documents for 2014 [23, 24]. For those supply items where there were no data in 2014, we collected data from 2011 to 2013 at PFSA [31, 32].
- Infrastructure cost: The cost of infrastructure was estimated using the direct allocation approach, based on the average client visits per day [25]. In fact, details of the number of rooms in the health facility, rooms used for PMTCT service, the presence of other integrated services provided at the PMTCT rooms, the average number of clients other than PMTCT clients and the average number of PMTCT clients at the rooms were collected. The information about the type of room, that is, whether the room was jointly used for both PMTCT service and maternal and health services or exclusively dedicated to PMTCT, was ascertained during field data collection. An estimated cost of health facility (hospital and health centres) construction was collected from the FMoH infrastructure directorate. The maintenance and utilities cost was also estimated in consultation with senior experts at the FMoH. The daily average cost of the infrastructure was calculated considering the data inputs: total cost of a building, working life years, annual discount rate, annual equivalent cost, inflation index, expected operation in months or days and average cost per day of operation. Together, the cost of infrastructure, maintenance and utilities per room were apportioned based on the average PMTCT client visits per day.
- Equipment cost: The different types of medical equipment (e.g. weighting scale for adults/infants, meters, blood pressure apparatus, stethoscopes, examination beds, chairs, tables, shelves, refrigerators, computers, etc.) used in the PMTCT service were identified, including year of procurement. The unit cost was collected from the PFSA equipment lists. For some of the equipment, where there were no price data from the PFSA, a local market assessment was conducted. The useful life was collected primarily from the Ethiopian government procurement documents. For those items where there was no monetary record, supplementary costing evidence was collected from the World Health Organization Choosing Interventions that are Cost-Effective (WHO-CHOICE) website [33]. For instance, to estimate the average cost of the weighting scale (for the infant), first, the year of purchase was reviewed and, second, the total estimated cost extracted from PFSA were considered. Finally, the annual equivalent cost and a breakdown by the number of days in operation were estimated. The cost of equipment for PMTCT was apportioned based on the number of PMTCT clients.
- Training cost: Details of relevant training received by staff were collected at the surveyed health facilities. A total of 12 PMTCT health professionals were interviewed and asked whether they had received PMTCT training during the past three years since the eMTCT strategy started. If they actually received formal training as per the PMTCT protocol [34] or the current draft guidelines, the total cost of training was collected from the RHBs databases. The annual training cost was calculated based on the number of training participants. The annual training cost was estimated per each health facility. Finally, the average cost of the training (per PPY) was apportioned based on annual PMTCT clients at the health facility.
- Programme management: According to the revised PMTCT guidelines and strategic plan documents [34, 35], programme management, monitoring and evaluation were the critical extra-facility inputs for improving quality and service harmonisation across the service package [34]. The amount of time spent and resources used for daily, weekly and quarterly review and for management meetings were collected and estimated at the surveyed health facilities. Additional data including per diem rates, allowances and incentives were also collected. Finally, at each health facility, the annual estimated cost attributed to the programme management was estimated and further apportioned to calculate the average cost per PPY.

CD4 count and EID services were considered in the PMTCT service packages. The different resources for providing CD4 and DBS (dried blood spot) testing were computed by applying a similar costing technique, notably apportioning labour cost based on time spent on providing the test and other shared costs on the basis of patients using CD4 counts. The resource allocated directly to CD4 count testing include the cost of inputs, such as consumables, used in this service.

### Sensitivity analysis

A sensitivity analysis in the estimation of national estimates was conducted using three scenarios: at the 57% current coverage, at the 75% coverage level and at 100% coverage level, although, caveat needs to be born in mind. The assumption of 100% coverage level might result in more than the costs obtained in sensitivity analyses due to possible diseconomies of scale that might arise from managing a large-scale response of such a magnitude.

### Ethical considerations

The research project was submitted and approved by the University of KwaZulu-Natal (UKZN) Biomedical Research and Ethics Committee (BREC REF 385/14) and the Federal Ministry of Health/ Ethiopian Public Health Institute (EPHI) Scientific and Ethical Review Office (SERO REF: 6.13/80). The data collection tool and Excel costing sheet were adapted from a similar costing survey in South Africa and from a malaria elimination costing sheet [36]. To refine the tool, it was piloted in two health facilities in Addis Ababa: Gandhi Memorial Hospital and Beletshachew Health Center.

## Results

In urban high HIV prevalence health facilities, the estimated cost of providing a PMTCT service per PPY ranged from ETB 6280 (USD 319) to ETB 21,620 (USD 1099). The mean USD per PPY was higher in hospitals than in health centres. The mean cost of providing a PMTCT service (per PPY) was ETB 13,782 (USD 701) with the highest cost of ETB 20,779 (USD 1056) and ETB 21,620 (USD 1099) recorded at HiwotFana Hospital and AFRTH hospital, respectively. In rurally located low HIV prevalence health facilities, on the other hand, the average cost per PPY ranged from ETB 4323 (USD 220) to ETB 7539 (USD 383) with a mean cost per PPY of ETB 5935 (USD 302). The cost variation was explained by the type and quantity of resource ingredients and by the type of health facilities (health centre, hospital centre). Most of the facilities in urban high HIV prevalence settings were hospitals (including HiwotFana and AFRTH), which were better equipped with different resources (labour, supplies and capital) relative to health facilities typically in rural settings. The detailed average cost per each health facility is described in Table 2. The details with respect to each resource input are explained in Table 3.

**Table 2:** Annual average cost per PPY in urban and rural surveyed health facilities

Urban health facilities	Average cost PPY (ETB)	Average cost PPY (USD)	Rural health facilities	Average cost PPY (ETB)	Average cost PPY (USD)
Teklehaimanot Health Center	6280	319	Atinago Health Center	5730	291
Soddo Health Center	8407	427	Chewaka Health Center	4322	220
HiwotFana Hospital	20,779	1056	Dedo Health Center	7539	383
DileChora Hospital	14,410	733	Hasange Health Center	6319	321
Bahir Dar Hospital	11,195	569	Kokossa Health Center	6104	310
AFRTH Hospital	21,620	1099	Toke Health Center	5598	285
Mean	13,782	701	Mean	5935	302
Stdev (SD)	6366	324	Stdev (SD)	1049	53

**Table 3:** Annual cost estimation of the PMTCT resource ingredients across the urban-rural health facilities

	Urban health facilities							Rural health facilities						
	Teklehaimanot Health Center	Soddo Health Center	HiwotFana Hospital	DileChora Hospital	Bahir Dar Hospital	AFRTH Hospital	Mean	Limuseka Health Center	Chewaka Health Center	Dedo Health Center	Hasange Health Center	Kokossa Health Center	Toke Health Center	Mean
Labour costs_ETB	1592	1207	4812	2934	1152	9987	3614	367	732	2483	416	1973	1268	1207
Labour costs_USD	81	61	245	150	59	508	184	19	37	126	21	100	65	61
Consumables_ETB	2862	2880	3152	2801	3525	3302	3087	1617	1911	2726	2704	3064	2950	2495
Consumables_USD	146	146	163	142	179	168	157	82	97	139	138	156	150	127
Equipment_ETB	194	19	733	434	287	196	311	980	57	518	1292	158	298	551
Equipment_USD	10	1	37	22	15	10	16	50	3	53	66	8	15	33
Infrastructure_ETB	1565	3918	13,393	7462	5924	8135	6733	2585	1623	1748	1826	908	596	1548
Infrastructure_USD	80	199	681	379	301	414	342	131	83	89	93	46	30	79
Training_ETB	66	384	409	780	307	0	324	181	0	64	81	0	486	135
Training_USD	3	20	21	40	16	0	17	9	0	3	4	0	25	7
Number of ANC-PMTCT pregnant women per year (2014)	2072	518	777	530	1036	600	922	259	520	500	775	510	260	470

### Cost components

In urban high HIV prevalence settings, the infrastructure cost accounted for the highest proportion of the total cost (48%) followed by labour (26%) and consumables (22%), while the consumables cost accounted for the highest proportion (42%) in rural low HIV prevalence health facilities followed by infrastructure and labour cost, which accounted for 26 and 20% of the estimated cost, respectively.

Of the listed consumables in rural areas, antiretroviral drugs and other drugs costs, including costs of Zidovudine (AZT) + Lamivudine (3TC) + Nevirapine (NVP), Isonicotinylhydrazide (INH) prophylaxis, cotrimoxazole, anti-bacterial drugs, NVP prophylaxis syrup and cotrimoxazole syrup (for the infants), comprised more than 80% of the total cost. The cost of labour does not rank second in rural area as it does in urban areas due to understaffing and relatively junior health care workers (who had a diploma or lower qualification) in rural setting.

Overall, the costs per PPY are higher in urban settings. Higher costs per PPY in urban settings are explained by first, higher cost of infrastructure, equipment and labour in urban areas and; secondly, the higher number of visits in urban areas as compared to the rural areas. Furthermore, the estimation of the average cost per PPY and not per visit implies higher cost per PPY in urban facilities, where relatively more frequent visits were recorded. The proportion of infrastructure cost varied from 25 to 60% in urban high HIV prevalence settings.

In both settings (urban high HIV prevalence and rural low HIV prevalence), the lowest proportion of costs (2%) was incurred for the training. According to the health workers interview report, there were health facilities that did not receive any formal PMTCT service training, or the trained staff had resigned and left the health facility. On the other hand, three resource items, namely consumables, labour and infrastructure contributed to more than 87% of the unit cost (per PPY). The detailed average cost estimation of the different PMTCT resource ingredients across the urban high HIV prevalence and rural low HIV prevalence settings is summarised in Table 3.

Labour and infrastructure costs accounted for 74% of the resources used for the PMTCT service provision. This can be explained by the higher cost of infrastructure such as buildings and their maintenance, in addition to relatively expensive experienced experts with many years of working experience, mainly in urban settings. The smallest proportion of the costs was estimated for equipment and training. The list of relevant resources (and calculated cost) used in the hospital and health centres is detailed in Additional files 1 and 2.

### Costing the PMTCT service packages

**Fig. 3:**
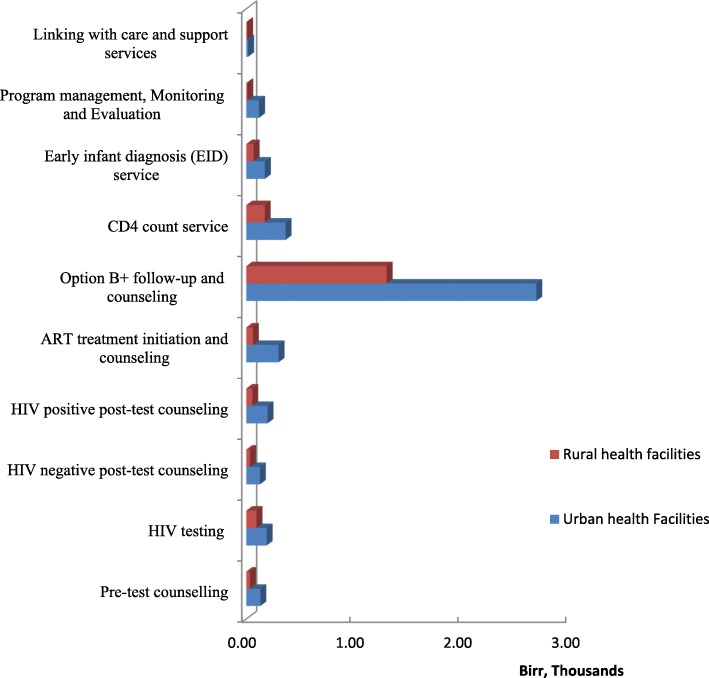
Cost of PMTCT service components in urban and rural health facilities

In the PMTCT service continuum, HIV/AIDS counselling and testing contributed 15 and 10% in urban high HIV prevalence and rural low HIV prevalence health facilities settings, respectively. Of the listed HIV/AIDS counselling and testing activities, 40 to 50% of this cost was spent on HIV testing and HIV-positive post-test counselling in each setting. The cost of HIV-negative post-test counselling accounted for 2 to 3% of the total cost (per PPY) in each setting. As shown in Fig. 3, antiretroviral treatment (option B+) follow-up and counselling contributed to the highest proportion of the cost (62% in urban high HIV prevalence health facilities and 71% in rural low HIV prevalence health facilities). The major component of these costs was the provision of antiretroviral treatment (option B+ drugs) for the mothers and infants, which constituted 50%of these costs in rural health facilities and 30% in urban health facilities. The higher cost is because the provision of ART treatment requires more frequent patient visits to the health facilities and thus more inputs. The CD4 count service and EID accounted for 8 and 4% of the average cost per PPY in urban and rural settings, respectively.

Programme management accounted for 3% of the unit cost (per PPY) in the urban high HIV prevalence facilities settings, but was a relatively lower proportion of the cost in rural low HIV prevalence facilities (i.e. 0.5%). The cost difference is due to the cost incurred by the top management teams at urban facilities for conducting service planning, monitoring and evaluation, as well as for frequent review meetings to improve service quality and efficiency.

### National estimates

**Fig. 4:**
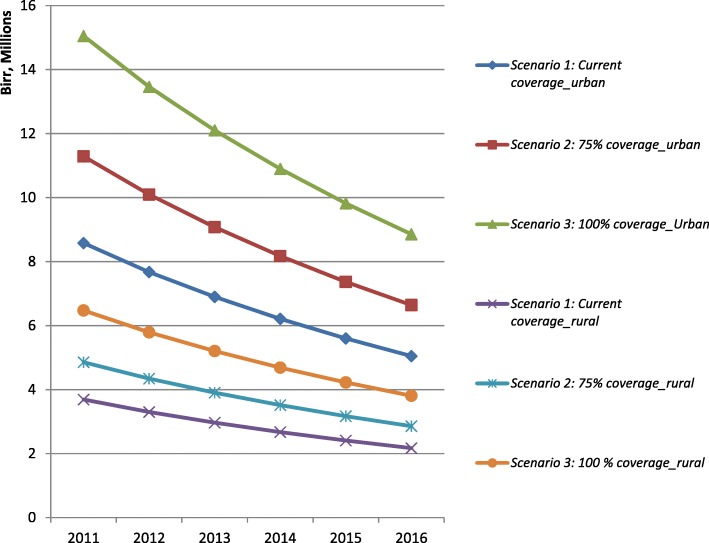
The national PMTCT service cost estimation in urban and rural settings, 2011 to 2016

At the current coverage level, the total cost of the PMTCT service at the base year, 2014, was estimated to be USD 6 million in urban settings. The corresponding cost for rural settings was estimated to be USD 3 million (Fig. 4). At 75% coverage, the national estimated total cost was USD 8 million and USD 4 million in urban and rural settings, respectively. If the service access improved to 100% universal coverage, the total cost of the service in urban areas would increase to USD 11 million, while the cost in rural areas would increase to USD 5 million. A caveat needs to be borne in mind here that the cost of 100% universal coverage could be higher due to diseconomies of scale that might arise from difficulties in implementation and management of the programme.

## Discussion

While local context costing evidence is relevant for healthcare planning, budgeting and cost-effectiveness analysis, it continues to be scarce in Ethiopia. This study was undertaken to conduct a costing exercises to fill the evidence gap by distinguishing the costing evidence of PMTCT in rural and urban settings. The costing analysis indicated that cost per PPY ranged from 4323 ETB (USD 220) in low HIV prevalence rural health facilities to 21,620 ETB (USD 1099) in high HIV prevalence urban health facilities. Estimates from similar studies in sub-Saharan African countries reported comparable average costs per PPY. A study by Touréet al. [37], for example, reported unit costs in the range of USD 203 to USD 1030 in Namibia and USD 94 to USD 342 in Rwanda. The studies in Namibia and Rwanda both applied an ingredient-based costing including HIV testing and counselling, CD4 cell count service, antiretroviral drugs, community-based activities, provision of medication postpartum, co-trimoxazole with prophylaxis and EID. In Rwanda, none of the surveyed health facilities had a CD4 count service, therefore, this was not captured in the costing estimation, while in Namibia the facilities costing included a CD4 count service. In addition, wages in Namibia were higher than in Rwanda. Similarly, our cost estimate in urban health facilities ranged from USD 319 to USD 1099.12. According to our analysis, urban health facilities were better equipped and resourced than rural facilities, which may be similar to Namibia’s health facilities. However, a relatively low unit cost was estimated in rural low HIV prevalence areas (USD 220 to USD 383), which is more comparable with the unit costs reported in Rwanda. In Ethiopia, Bikilla et al. [16] reported a unit cost (per PPY) of USD 235 and USD 29 for ART outpatient and inpatient care, respectively. This finding was also comparable to the low HIV prevalence rural health facilities costing report, which ranged from ETB 4323 (USD 220) to ETB 7539 (USD 383).

Our analysis also unveiled a wide variation of costing estimates between urban and rural settings, which was explained by the difference in the type and quantity of resources (labour, consumables and infrastructure) used across the high and low HIV prevalence and urban-rural settings, as well as the type of health facilities sampled for the costing analysis. Most of the facilities in urban high HIV prevalence settings were tertiary level hospitals (four out of the six health facilities) that were better equipped in terms of resources as compared to the rural health facilities that were less equipped in terms of labour, equipment and infrastructure. The higher costs of infrastructure and other inputs into PMTCT services in urban settings meant that more patients’ visits resulted in no reduction of cost per PPY and this yielded no economies of scale. In fact, previous studies have also used the same argument to explain the cost differences. Desmond et al. [36], for example, reported that HIV prevalence and resource distribution were the main determining factors affecting the wide cost variation across well-resourced sites in Western Cape and Limpopo provinces and low HIV prevalence and poorly-resourced provinces in KwaZulu-Natal and Free State provinces in South Africa. In another study, Desmond and Boyce [38] also assessed the financial and economic cost differences in the rural PMTCT sites in Eastern Cape, South Africa and found that staff time was the main cost contributor. However, our study identified medical consumables (in rural low HIV prevalence sites) as the main cost driver. The difference was because our study considered the new PMTCT Option B+ lifelong treatment, while the study by Desmond and Boyce [30] considered the single-dose nevirapine and cotrimoxazole antiretroviral prophylaxis drug regimens.

A literature review by Touréet al. [37] reported 6 to 15% of the estimated cost to be attributed to the CD4 count service. Similarly, our analysis estimated 8% of the unit cost (per PPY) as attributable to the CD4 counts-related service. While this study reports similar costing results for some of the PMTCT service packages, it also points out a higher unit cost estimate for infrastructure that comprised 48% of the unit cost of the PMTCT service (per PPY). This may be attributed to the low PMTCT service uptake and underutilisation of the service by antenatal care-attending HIV-positive pregnant women in the country [1, 11]. According to UNAIDS [1], Ethiopia is listed as one of the 22 targeted countries achieving less than 50% coverage in 2012, which, as already noted, is very low as compared to 90% coverage in Ghana, Zambia, Namibia and Botswana. For instance, the 2011/12 Ministry of Health Annual Performance Report [39] indicated that a total of 9775 HIV-positive mothers received ART treatment in the 2004 Ethiopian fiscal year (EFY), which is far below the target set for the year (i.e. 38,405).

In the current effort to eliminate new HIV infections and to support the current UHC agenda, the relevance of having context-specific costing data for PMTCT and analysing the differences between urban and rural health facilities is critical. However, Ethiopia in particular, has faced a lack of critical evidence on the cost of PMTCT programmes [15–17]. Earlier costing studies have analysed the cost and financial requirements for the scale-up of HIV/AIDS services in limited parts of the country. However, neither of these studies assessed the PMTCT service taking into account HIV heterogeneity across urban-rural health facilities, which is believed to be important for improving health care delivery efficiency [40, 41], performance management [42], future improvement opportunities [40] and identifying the potential for cost containment and reduction [43]. This study contributes to the literature by providing this evidence.

Although the analyses highlighted the unit cost and national costing difference in urban high HIV prevalence versus rural low HIV prevalence settings, the study has some important shortcomings. The reported unit cost results do not necessarily reflect the level of efficiency, as the outcome data over a period of time was not analysed. This study only provided a snapshot of costing results across the heterogeneous HIV prevalence and urban-rural settings. However, considering these findings and complementary panel data, future research may report the technical or allocative efficiency differences across these settings. In addition, we considered the estimated unit cost result per PPY from the two settings (urban high HIV prevalence and rural low HIV prevalence) to estimate the national PMTCT service cost from the health care provider perspective. However, the facilities were not randomly selected and costing estimates derived from nationally representative sampled health facilities might be required.

## Conclusions

The current global economic downturn and reduction in international finance requires substantial costing evidence to inform health care policy and programme management decisions. This information is particularly critical in countries with heterogeneous HIV prevalence countries, such as Ethiopia. Following a comprehensive costing analysis in this study, the finding is that resources used for PMTCT service packages varied across health facilities and HIV prevalence contexts. Furthermore, providing PMTCT service in the high HIV prevalence, urban health facilities costs more than in the rural facilities. Context-specific costing appears vital to provide locally sensitive evidence for health service management and priority setting.

## Additional files

Additional file 1: Health center resource lists and computed average unit/ annual cost across the different inputs in Ethiopia (DOCX 23 kb)

Additional file 2: Hospital resource lists and computed average unit/ annual cost across the different inputs in Ethiopia (DOCX 25 kb)

## Abbreviations

AFRTH: Armed Forces Referral and Teaching Hospital
ANC: Antenatal care
ART: Antiretroviral therapy
DBS: Dried blood spot
EFY: Ethiopian fiscal year
EID: Early infant diagnosis
eMTCT: Elimination of mother-to-child transmission
EPHI: Ethiopian Public Health Institute
ETB: Ethiopian birr
FHAPCO: Federal HIV/AIDS Prevention and Control Office
FMoH: Federal Ministry of Health
HMIS: Health Management Information System
IMF: International Monitoring Fund
MDGs: Millennium development goals
NVP: Nevirapine
PFSA: Pharmaceutical Fund and Supply Agency
PMTCT: Prevention of mother-to-child transmission of HIV/AIDS
PPY: Per pregnant woman-infant pair per year
RHBs: Regional Health Bureaus
SDG: Sustainable development goals
SPSS: Statistical Package for the Social Sciences
UHC: Universal health coverage
UKZN: University of KwaZulu-Natal
USD: United States dollar
WHO-CHOICE: World Health Organization Choosing Interventions that are Cost-Effective
