# Supplementary material for: Assessing the cost of providing a prevention of mother-to-child transmission of HIV/AIDS service in Ethiopia: urban-rural health facilities setting
Source: BMC Health Serv Res. 2019 Mar 6;19:148. doi: 10.1186/s12913-019-3978-4 (PMC6404341; doi:10.1186/s12913-019-3978-4)
Supplement: Supplementary file 2 — Hospital resource lists and computed average unit/ annual cost across the different inputs in Ethiopia (DOCX 25 kb) [file 12913_2019_3978_MOESM2_ESM.docx]

**Additional file 2:** Hospital resource lists and computed average unit/ annual cost across the different inputs in Ethiopia

| **Hospital resources** | | **Unit cost** | |
| --- | --- | --- | --- |
| **1** | **Labor cost** | **Average salary  per day (ETB)** | **Average salary per day (USD)** |
|  | Physician (specialist) | 833.51 | 42.37 |
|  | Physician (specialist) | 833.51 | 42.37 |
|  | Health officer | 523.90 | 26.63 |
|  | Health officer | 523.90 | 26.63 |
|  | Senior nurse officer | 354.73 | 18.03 |
|  | Diploma nurse | 127.71 | 6.49 |
|  | Diploma nurse | 127.71 | 6.49 |
|  | Diploma nurse | 127.71 | 6.49 |
|  | Beginner diploma nurse | 112.50 | 5.72 |
|  | Mother support group(MSG) | 36.73 | 1.87 |
|  | Mother support group(MSG) | 36.73 | 1.87 |
|  | Mother support group(MSG) | 36.73 | 1.87 |
|  | Mother support group(MSG) | 36.73 | 1.87 |
|  | Mother support group(MSG) | 36.73 | 1.87 |
| **2** | **Supplies** | **Cost  per unit (ETB)** | **Cost  per unit (USD)** |
|  | Disposable gloves | 66.68 | 3.39 |
|  | HIV testing kits: KHB | 576.35 | 29.30 |
|  | Lancet | 68.85 | 3.50 |
|  | HIV testing kits: Statpack | 666.83 | 33.90 |
|  | HIV testing kits: Unigold | 941.23 | 47.85 |
|  | Alcohol | 69.04 | 3.51 |
|  | Cotton | 23.01 | 1.17 |
|  | Capillary tube | 179.00 | 9.10 |
|  | DBS kits | 1121.22 | 57.00 |
|  | Humidity | 961.89 | 48.90 |
|  | ART drug regimen: TDF + 3TC + EFV | 196.71 | 10.00 |
|  | INH Prophylaxis | 64.72 | 3.29 |
|  | Panadol/ Paradox | 157.56 | 8.01 |
|  | Cotrimoxazole | 391.64 | 19.91 |
|  | Iron folate | 58.62 | 2.98 |
|  | Cotrimoxazole syrup (for the infants) | 8.85 | 0.45 |
|  | NVP Prophylaxis (for the infants) | 38.36 | 1.95 |

| **3** | **Equipment** | **Annual cost (ETB)** | **Annual cost (USD)** |
| --- | --- | --- | --- |
|  | Weighting scale (2) | 1626.91 | 82.71 |
|  | Height scale (1) | 813.46 | 41.35 |
|  | MUAC (2) | 23.75 | 1.21 |
|  | BP apparatus (1) | 290.94 | 14.79 |
|  | Stetoscope (2) | 243.44 | 12.38 |
|  | Shelf big size (1) | 999.74 | 50.82 |
|  | Examination bed (1) | 843.15 | 42.86 |
|  | Screen (1) | 504.70 | 25.66 |
|  | Table (3) | 1048.94 | 53.33 |
|  | Chairs (4) | 999.74 | 50.82 |
|  | Guest chair (3) | 749.81 | 38.12 |
|  | Weighting scale (for the infant) | 486.89 | 24.75 |
| **4** | **Medicines** | **Unit cost  per person (ETB)** | **Unit cost  per person (USD)** |
|  | **For the pregnant women** |  |  |
|  | ART drug regimen: TDF + 3TC + EFV | 2356.22 | 119.78 |
|  | INH Prophylaxis | 123.70 | 6.29 |
|  | Cotrimoxazole | 149.72 | 7.61 |
|  | Panadol/ Paradox | 100.39 | 5.10 |
|  | Iron folate | 5.60 | 0.28 |
|  | **For the infant** |  |  |
|  | NVP Prophylaxis (for the infants) | 40.73 | 2.07 |
|  | Cotrimoxazole syrup (for the infants) | 112.80 | 5.73 |
| **5** | **Transportation costs** | **Unit cost (ETB)** | **Unit cost (USD)** |
|  | Disposable gloves | 9.49 | 0.48 |
|  | HIV testing kits: KHB | 82.06 | 4.17 |
|  | Lancet | 9.80 | 0.50 |
|  | HIV testing kits: Statpack | 94.95 | 4.83 |
|  | HIV testing kits: Unigold | 94.12 | 4.79 |
|  | Alcohol | 9.83 | 0.50 |
|  | Cotton | 3.28 | 0.17 |
|  | Capillary tube | 25.49 | 1.30 |
|  | DBS kits | 112.12 | 5.70 |
|  | Humidity | 0.00 | 0.00 |
|  | ART drug regimen: TDF + 3TC + EFV | 20.89 | 1.06 |
|  | INH Prophylaxis | 6.87 | 0.35 |
|  | Panadol/ Paradox | 16.73 | 0.85 |
|  | Cotrimoxazole | 41.59 | 2.11 |
|  | Iron folate | 6.22 | 0.32 |
|  | Cotrimoxazole syrup (for the infants) | 0.94 | 0.05 |
|  | NVP Prophylaxis (for the infants) | 4.07 | 0.21 |
|  |  |  |  |
| **6** | **Infrastructure costs** | **Unit cost per  day of operation (ETB)** | **Unit cost per  day of operation (USD)** |
|  | Hospital (construction and maintenance costs) | 208866.77 | 10618.27 |
|  | Medical surgical | 14236.29 | 723.74 |
|  | Pediatrics | 23388.20 | 1189.00 |
|  | MCH | 53284.41 | 2708.85 |
|  | Gyn | 22371.32 | 1137.30 |
|  | Gyn-Obs | 8135.03 | 413.56 |
|  | Neonate | 16270.05 | 827.13 |
|  | ICU - Surgical | 8135.03 | 413.56 |
|  | ICU- Medical | 8135.03 | 413.56 |
|  | OPD | 40675.13 | 2067.82 |
|  | Admin rooms | 14236.29 | 723.74 |
|  | ANC big rooms 1 | 14236.29 | 723.74 |
|  | ANC room 2 | 14236.29 | 723.74 |
|  | ANC room 3 | 14236.29 | 723.74 |
|  | PMTCT/DBS room 1 | 406.75 | 20.68 |
|  | ART room 1 | 10168.78 | 516.96 |
| **7** | **Training costs** | **Annual cost (ETB)** | **Annual cost (USD)** |
|  |  |  |  |
|  | Training costs | 16200.00 | 823.57 |
|  |  |  |  |
| **8** | **Program management** | **Annual cost (ETB)** | **Annual cost (USD)** |
|  |  |  |  |
|  | Program management (M&E) cost | 17574.24 | 893.43 |
